# Medical student attitudes and educational interventions to prevent neurophobia: a longitudinal study

**DOI:** 10.1186/s12909-017-1055-4

**Published:** 2017-11-21

**Authors:** Lisa Shiels, Pratish Majmundar, Aleksander Zywot, John Sobotka, Christine S. M. Lau, Tuula O. Jalonen

**Affiliations:** 1 Department of Physiology and Neuroscience, St. George’s University School of Medicine, St. George’s, Grenada; 2 Department of Surgery, Morristown Medical Center, Morristown, NJ USA

**Keywords:** Neuroscience, Caribbean, Team based learning, Case based teaching, Problem based learning

## Abstract

**Background:**

With an aging American population, the burden of neurologic disease is intensifying and the decline in neurology residents and practicing neurologists is leaving these patients helpless and unable to find care. ‘Neurophobia’, a chronic illness that begins early in medical school, has been identified as a cause for the low number of neurology residents.

**Methods:**

A longitudinal study surveyed medical students at the beginning of their first year (M1) and then again at the beginning of their second year (M2). Three neuroscience educational interventions were studied: team based learning (TBL), case based teaching (CBT), and problem based learning (PBL). Participants provided self-reported neurophobia levels, attitudes about neuroscience, and the effectiveness of educational interventions.

**Results:**

A total of 446 students during M1 and 206 students during M2 participated in the survey. A significant change in self-reported neurophobia (*p* = 0.035) was observed from 19% in M1 to 26% in M2. Neuroscience knowledge and confidence managing a neurologic condition also significantly increased (*p* < 0.001 and *p* = 0.038 respectively). Perceived interest, difficulty, and desire to pursue a career in neuroscience did not a change significantly. Majority of students perceived CBT (76%), TBL (56%), and PBL (66%) beneficial. Only CBT demonstrated a statistical difference (*p* = 0.026) when stratified by self-reported change in neurophobia.

**Conclusion:**

An increase in neurophobia after completing a neuroscience was observed but the prevalence rate of 26% was lower than previous studies. Knowledge about neuroscience increased significantly and educational interventions were considered beneficial by students. Thus, interventions that increase knowledge and decrease neurophobia can lead to an increase in students pursuing neurology residencies.

## Background

With an aging American population, the burden of neurologic disease is intensifying through an increase in both incidence and prevalence [1]. The decline in medical students pursuing residencies in neuroscience ultimately leads to less practicing neurologists, leaving these patients helpless and unable to find care [2–4].

‘Neurophobia’, a term first coined by Jozefowicz in 1994, describes medical students’ fear of neurology [2]. It is a chronic illness that begins early in medical school [4]. Physicians and medical students alike often state that neurology is the most difficult subject in the medical school curriculum, and that their knowledge about the subject matter is limited, leading to a lack of confidence in managing neurology patients [5–7].

The number of new neurologists is growing at a much slower pace compared to other specialties, in both the United States (US) and Europe [8]. From 2010 to 2015, the growth in both the number of students applying for and the number of programs offering residencies in the US in neurology (+1.3%) has paled in comparison to surgery (+4.6%), psychiatry (+4.9%), family medicine (+11.6%), and internal medicine (+23.4%) [9].

Since Jozefowicz’s original observation, researchers have been studying neurophobia to identify the cause and provide a cure. Schon et al. [5] designed a clinical specialty attitudes survey with the aim of identifying attitudes and factors associated with neurophobia . Medical students and residents who participated in the survey reported that their knowledge for neurology was less than for other specialties and they perceived neurology to be the most difficult specialty compared to cardiology, endocrinology, gastroenterology, geriatrics, respiratory medicine, and rheumatology [5]. Neurophobia was therefore postulated to, at least in part, be caused by the perceived difficulty of neurology and students’ poor knowledge of the subject [5]. Building on the findings of Schon et al., several studies integrated case based teaching (CBT) with the basic science portion of the neuroscience curriculum resulting in increased self-reported neuroscience knowledge and in turn decreased self-reported neurophobia levels [1, 6, 10]. Ridsdale et al. [4] employed team based learning (TBL) and demonstrated an improvement in student confidence in dealing with neurologic cases. Giles [11], Wiles [12] and others then highlighted the benefits of interactive learning and using problem based learning (PBL) to improve students’ understanding of neuroscience [4, 11–13].

Previous studies have implemented educational interventions such as CBT, TBL, and PBL, and have shown an increase in self-reported neuroscience knowledge. The aim of this study is therefore to assess the neurophobia level of incoming students, its evolution during the first year of medical school, and the perceived benefit of CBT, TBL, and PBL.

## Methods

### Participants and design

A longitudinal study was conducted following the 2014 incoming class of medical students, from the start of medical school to the completion of the neuroscience portion of the curriculum, at St. George’s University (SGU) School of Medicine in Grenada, West Indies. SGU’s medical school curriculum is a 4-year program with 2 years of basic science and 2 years of clinical clerkships. SGU utilizes a subject based approach during the basic science portion comprising the first 2 years. Year one is divided into two terms with first term courses in Anatomy, Histology and Biochemistry, and second term courses in Neuroscience, Physiology, Genetics and Immunology. Year two consists of three terms with a course in Behavioral Sciences in term three, Pathology, Microbiology, and Clinical Skills in term four, and Pathophysiology, Pharmacology, and an Advanced Clinical Skills in term five.

The neuroscience class at SGU was taught three times a week by three professors who rotated teaching the material based on their specialty. The course consisted of seven modules: neuroanatomy, cellular neuroscience, neurodevelopment, sensory systems, motor systems, regulatory systems, and complex brain functions. The course was taught in a lecture hall sufficiently large to accommodate the entire class. Attendance was mandatory for all students. Two exams were administered, a midterm exam after the first three modules and a final exam at the end of the course.

Approval to conduct the study was obtained from the SGU Institutional Review Board (IRB #14037) and informed consent forms were obtained from all participants. Student participation was voluntary and had no effect on student grades, performance, or evaluation. All first semester medical students were invited to participate in the survey (M1). Students were again invited to participate in the survey at the start of their second year following completion of the neuroscience class (M2). Students were grouped as either pre-neuroscience year one students (M1) or post-neuroscience year two students (M2). Cook et al. [14] established standards for medical education submissions which were used to guide data analysis, interpretation of results, and the reporting of findings.

### Educational interventions

Three educational interventions that previous studies identified to be beneficial in improving neuroscience knowledge and decreasing neurophobia were studied: TBL, CBT, and PBL [4–6, 8, 10–13, 15, 16].

#### TBL

The objective of TBL is to have participants solve problems by applying the knowledge they recently gained. Through self and team learning, students reinforce course concepts. TBL consists of four core steps [17, 18].

TBL begins with the formation of permanent teams. Ideally teams should be created by instructors with a diverse group of 5–7 students per team [17]. Next student knowledge is assessed with the Readiness Assurance Test (RAT) [17]. Several days prior to the start of the TBL session students are provided preparatory material and then at the start of the session students’ comprehension and knowledge are evaluated using the RAT. The instructor then reviews the results of the RAT with the class, clarifying any misconceptions that exist. The third step of TBL is student participation in team based learning activities [17]. These activities are designed to promote group discussion, critical thinking, and knowledge application. The fourth and final step is the completion of an in-class assessment [17]. This process serves to both reinforce the material learned and to create accountability for each team member. Upon completion feedback is provided to once again aid in clarifying misconceptions.

At St. George’s University, a total of 10 two-hour neuroscience TBL sessions were offered evenly throughout the semester. At the start of the semester each student was assigned to a team by the course professor in order to create a fair distribution of knowledge and diversity. Each team was comprised of about six students and lead by a student facilitators. Clinical experts and course professors were available and served as expert consultant if requested. Learning objects and resources for each session were provided to students the week prior to the session. Students completed a self-assessment at the start of the session. At the end of every session each student, in collaboration with his or her team members, completed a short session examination. Feedback was provided and explanations were offered to students. Students received a grade for participation in each session and for completion of the end-of-session examination. Students who missed more than 20% of these sessions automatically failed the course.

#### CBT

CBT aims to make teaching student focused by having students actively process information in an attempt to solve a problem [19–21]. This form of learning forces students to process information, modify methods of thinking, and combine new knowledge with previous knowledge. In CBT, basic sciences and clinical sciences are integrated, making learning more effective and interesting for students.

A clinical case is first presented to students, providing basic patient history [21]. Next either questions follow, testing basic science and clinical knowledge, or a clinical change is presented and students are asked to reason through the processes of change [21]. Throughout the case a professor or clinical expert is present to guide the discussion, student reasoning, and clarify any misunderstandings.

Sixteen one-hour CBT sessions were held throughout the neuroscience semester at SGU. Four sessions were held during the first half of the semester and 12 were held in the second half. This uneven distribution was due to more clinical based lectures were held during the second half of the semester. CBT sessions were intended to facilitate the application of clinically relevant knowledge gained during the preceding lectures and to help students develop their skills in critical clinical thinking and reasoning. Learning objectives were provided to students in advance and students were encouraged to review relevant lecture material. Case discussions were held in the lecture hall were student discussions were guided by faculty. For each case, students discussed the case history, reviewed examination findings and techniques, and rationalized through the diagnosis.

#### PBL

PBL is a simpler learning strategy in which students are given a partial case and are guided in solving a problem that contains some degree of foreign concepts [20, 22]. In PBL, similar to TBL, students are divided into groups and attempt to solve problems as a team [17]. Unlike TBL however, in PBL students solve problems to identify knowledge gaps [17]. Students then undertake self-directed studying to remedy areas of weakness.

At SGU, 59 one-hour neuroscience lectures were offered to students with the last 10–15 min of every lecture reserved for a PBL session. Questions presented to students were designed to expand on the material presented in the previous lecture. Faculty were present to guide discussion, explain concepts if necessary, and aid students in designing focused learning objectives.

### Survey instrument

The survey instrument was based upon Schon et al.’s original questionnaire, which has since been implemented in multiple studies [5–7, 13, 23, 24]. A consortium of individuals at SGU from the biostatistics, psychology, and neuroscience departments met to design the questionnaire. The questionnaire contained three parts. Parts one and two were based on Schon et al.’s questionnaire and a third part was added to survey attitudes about educational intervention attitudes.

The first part of the questionnaire (Q1–8) collected minimal demographic information including sex, age, and current grade point average. Several questions then asked about educational background, previous neuroscience exposure, and learning preferences.

In part two of the questionnaire (Q9–14), the level of perceived neurophobia was assessed. Neurophobia was defined as medical students’ fear of neuroscience and directly measured by having respondents rank their agreement with the following statement “I have an aversion to neuroscience”.

Six questions from Schon et al.’s questionnaire were used to assess self-reported attitudes about neuroscience [5]. These questions evaluated: interest in neuroscience, knowledge in neuroscience, difficulty of neuroscience, confidence in managing a clinical case pertaining to neuroscience, and the desire to pursue a career in neuroscience. A 5-Point Likert scale was used to grade responses.

Part three (Q15-17) was only administered to M2 students and comprised of three questions focused on the educational interventions. A 5-Point Likert scale was used to grade responses.

### Data collection and outcomes

The survey questionnaire was administered twice to the same cohort of students, initially at the beginning of their first year of medical school (M1) and again when the students started their second year of medical school (M2). Parts one and two of the questionnaire were administered twice, during M1 and M2. Part three was only administered during M2.

Surveys were administered to the entire class with no exclusion based on age, sex, race, or prior educational experience. Student responses were submitted electronically and all responses were treated as confidential. Upon collection a unique electronic ID was assigned to participants which allowed for tracking students during the study. Consent to participate was provided on both occasions and participation was not dependent on a student answering all survey questions. Students who failed to answer to the question about neurophobia were excluded from the study.

The primary outcome was change in the level of self-reported neurophobia between M1 and M2. Neurophobia was defined as medical students’ fear of neuroscience and considered present when students answered positively to the statement “I have an aversion to neuroscience”.

Secondary outcomes were effectiveness of the educational interventions (TBL, CBT, and PBL) and changes in self-reported attitudes about neurology between M1 and M2.

### Statistical analysis

The data collected were coded and analyzed using Statistical Package for Social Sciences v. 20 (SPSS Inc. Chicago, IL, USA). Individual student responses were compared between groups using frequencies and Chi-square analysis. Matched analysis was conducted using a Wilcox Signed Rank test. A two-tailed *p*-value of <0.05 was considered statistically significant.

## Results

A total of 446 students participated in the pre-neuroscience survey (M1) and 206 in the post-neuroscience survey (M2) (Table 1). A matched cohort, comprised of only students that answered both surveys, had a total of 150 respondents. Matched and unmatched cohorts had similar distributions in demographics and self-reported attitudes (Table 1).

**Table 1:** Demographic characteristics participating medical students

	Unmatched		Matched
	M1 frequency (%)	M2 frequency (%)	Frequency (%)
	*N* = 446	*N* = 206	*N* = 150
Gender			
Male	196 (49%)	61 (50%)	73 (51%)
Female	204 (51%)	61 (50%)	71 (49%)
Age			
< 23	24 (6%)	8 (5%)	9 (6%)
23–28	326 (78%)	140 (81%)	115 (80%)
> 28	66 (16%)	25 (14%)	20 (14%)
Educational training			
High school	11 (3%)	3 (2%)	3 (2%)
Undergraduate degree	280 (67%)	116 (67%)	96 (68%)
Graduate degree	127 (30%)	51 (31%)	42 (30%)
Neuroscience familiarity			
No exposure	117 (27%)	36 (18%)	45 (30%)
School/Work exposure	213 (48%)	129 (65%)	71 (48%)
Neuroscience coursework	111 (25%)	35 (18%)	32 (22%)

*Abbreviations*: *M1* year one (pre-neuroscience) medical students, *M2* year two (post-neuroscience) medical students, *N* number of participants

### Demographic characteristics

In the matched cohort, the majority of students (80%) were 23–28 years of age with 51% males and 49% females. Most students had previous exposure to neuroscience with 48% reporting school or work experience and 22% taking neuroscience courses. The unmatched cohort had a similar distribution. In the unmatched cohort, there was no significant difference between the M1 and M2 groups in baseline demographic factors including age (*p* = .901), gender (*p* = .888), or educational background (*p* = . 963).

### Effect of a neuroscience course on student attitudes

Self-reported neurophobia levels and neuroscience attitudes were compared between M1 and M2 responses in the matched cohort (Table 2). The median response for neurophobia in M1 and M2 was a 3/5 on the Likert scale (neither agree nor disagree) but M2 had an increased frequency of “strongly agree” (11%) and “agree” (15%) responses compared to M1 (7 and 12% respectively). A Wilcoxon signed-rank test demonstrated a significant difference in self-reported neurophobia (*p* = 0.035).

**Table 2:** Wilcox signed-rank analysis of self-reported neurophobia and neuroscience attitudes in matched M1 and M2 responses

	M1 frequency (%)	M2 frequency (%)	*p*-value
Interest			0.327
None	21 (15%)	17 (17%)	
Slight	26 (18%)	11 (11%)	
Somewhat	40 (28%)	22 (22%)	
Moderate	36 (25%)	33 (33%)	
Extreme	19 (13%)	18 (18%)	
Knowledge			0.000
None	37 (27%)	8 (9%)	
Fair	53 (39%)	15 (16%)	
Good	32 (23%)	39 (43%)	
Very good	12 (9%)	24 (26%)	
Excellent	3 (2%)	5 (5%)	
Difficulty			0.057
Very difficult	35 (24%)	25 (27%)	
Difficult	71 (49%)	29 (31%)	
Neutral	34 (23%)	30 (32%)	
Easy	1 (1%)	6 (6%)	
Very easy	4 (3%)	3 (3%)	
Confidence			0.038
None	65 (46%)	29 (26%)	
Slight	41 (29%)	51 (46%)	
Somewhat	26 (18%)	29 (26%)	
Moderate	5 (4%)	2 (2%)	
Extreme	5 (4%)	1 (1%)	
Pursue career			0.063
Extremely unlikely	31 (22%)	38 (28%)	
Unlikely	35 (25%)	40 (30%)	
Neutral	59 (42%)	43 (32%)	
Likely	10 (7%)	8 (6%)	
Extremely likely	7 (5%)	5 (4%)	
Neurophobia			0.035
Strongly disagree	35 (23%)	20 (13%)	
Disagree	32 (21%)	44 (29%)	
Neither	61 (41%)	46 (31%)	
Agree	14 (9%)	23 (15%)	
Strongly agree	8 (5%)	17 (11%)	

*Abbreviations*: *M1* year one (pre-neuroscience) medical students, *M2* year two (post-neuroscience) medical students

Perceived interest in neuroscience did not a change between M1 and M2. The median response in M1 and M2 cohorts was 4/5 on the Likert scale (moderate) and the Wilcoxon signed-rank test in the matched cohort did not find a significant difference (*p* = 0.327).

During M2 the median response for neuroscience knowledge was a 3/5 on the Likert scale (good), which was an increase from the median response of 2/5 on the Likert scale (fair) in M1. The Wilcoxon signed-rank test showing a significant change (*p* < 0.001). Confidence in treating a neurologic condition also demonstrated a significant change (*p* = 0.038). Neuroscience was perceived equally difficult during M1 and M2 with a median response of 2/5 (difficult) by both groups with no significant change in responses. Students’ desire to pursue a career in neuroscience was also did no demonstrate a significant change between M1 and M2.

### Outcomes of educational interventions

Most students in the matched cohort reported that each educational intervention implemented was beneficial in assisting with neuroscience studies (Table 3). The median response was 4/5 (agree, 45%) for CBT, 4/5 (agree, 40%) for TBL, and 4/5 (agree, 40%) for PBL. Chi-square analysis compared the perceived benefit of each educational intervention stratified by the change in self-reported neurophobia levels from M1 to M2 (Table 3). CBT demonstrated a statistical difference (*p* = 0.026), while TBL (*p* = 0.623) and PBL (*p* = 0.425) did not.

**Table 3:** Chi-square analysis of perceived effectiveness of educational interventions stratified by change in self-reported neurophobia from M1 to M2 in a matched cohort of medical students

	Decreased neurophobia	No change in neurophobia	Increased neurophobia	Total	*p*-value
CBT					0.026
Strongly disagree	2 (5%)	1 (2%)	0 (0%)	3 (3%)	
Disagree	6 (15%)	2 (4%)	3 (10%)	11 (9%)	
Neither	3 (8%)	3 (6%)	8 (26%)	14 (12%)	
Agree	19 (49%)	20 (42%)	14 (45%)	53 (45%)	
Strongly agree	9 (23%)	22 (46%)	6 (19%)	37 (31%)	
TBL					0.623
Strongly disagree	3 (9%)	5 (11%)	2 (6%)	10 (9%)	
Disagree	5 (15%)	3 (7%)	3 (10%)	11 (10%)	
Neither	11 (32%)	9 (20%)	7 (23%)	27 (25%)	
Agree	10 (29%)	18 (41%)	16 (52%)	44 (40%)	
Strongly agree	5 (15%)	9 (20%)	3 (10%)	17 (16%)	
PBL					0.425
Strongly disagree	2 (5%)	4 (7%)	1 (3%)	7 (5%)	
Disagree	6 (15%)	5 (9%)	7 (19%)	18 (14%)	
Neither	10 (24%)	6 (11%)	4 (11%)	20 (15%)	
Agree	15 (37%)	22 (40%)	16 (44%)	53 (40%)	
Strongly agree	8 (20%)	18 (33%)	8 (22%)	34 (26%)	

*Abbreviations*: *CBT* case based teaching, *TBL* team based learning, *PBL* problem based learning

## Discussion

Neurophobia, defined as a medical student’s fear of neuroscience, is an affliction they will carry as a practicing physician and can therefore have long-term implications on patient care [2, 4]. Although the cause of neurohobia is multifactorial, studies have identified students’ lack of neuroscience knowledge as an associated factor [6]. Improving the medical educational curriculum is therefore considered the frontline intervention for preventing and treating neurophobia [6].

In this longitudinal survey, student’ self-reported attitudes towards neurology were measured and linked to self-reported neurophobia. Results showed a small but significant increase in neurophobia after exposure to the neuroscience course, providing further evidence that a medical school’s neuroscience education alters students’ perception of neurology.

Jozefowic’s initial paper (1994) stated that 50% of students experienced neurophobia [2]. Follow-up studies identified neurophobia rates ranging from 18–47% [10, 25, 26]. Fantaneanu et al. [27] examined the subject further and found 32% of students neurophobic of academic neuroscience and 24% neurophobic of clinical neuroscience. The prevalence rate of 26% (agree or strongly agree) at SGU falls in the lower end of this spectrum.

Factors associated with neurophobia were compared to those in previously published values. Interest in neuroscience 51% during M2 (moderate or extreme) was similar to previous studies such as Sanya et al. (2010) who found that 49% of students were interested in neurology [28]. At SGU, knowledge about neuroscience increased after students completed the course from 11% in M1 (very good or excellent) to 31% in M2. Although this was a significant increase, studies such as Schon et al. have demonstrated that students consistently report having the lowest amount of knowledge about neuroscience compared to all other specialties examined [5]. Neuroscience was perceived to be less difficult at SGU compared to previous studies, with only 58% of students during M2 reporting the subject as a difficult or very difficult. Abulaban et al. for instance reported that 86% of students considered the subject matter difficult [29].

Though the neurophobia prevalence rate of 26% at SGU is lower than most published studies, it does continue the trend of increasing neurophobia levels with increased years of medical education and exposure to the medical school neuroscience course [27]. Previous studies on neurophobia have shown that educational interventions can improve students’ knowledge of neurology and serve as a protective factor against neurophobia [1, 4, 6, 8, 10, 15]. The lower than expected level of neurophobia could therefore be in part due to the educational interventions implemented.

Although all educational interventions were shown to be beneficial to the majority of the class, several limitations inhibit this study’s ability to generalize results. First, this study did not have a separate control group, which limits the ability to examine the effectiveness of each educational intervention independently. It therefore becomes difficult to separate the impact of the neurology course and the impact of educational interventions on the level of neurophobia. Furthermore, the neurology course at SGU is different from neurology courses in other universities and the effect of SGU’s neuroscience course on neurophobia and students’ attitudes differs. This study focused on first and second year students instead of third or fourth year clinical clerkship students, who are more focused on selecting a specialty for residency. Lastly a precise instrument to measure neurophobia has yet to be designed and validated, thus making it difficult to compare between reported study findings.

## Conclusions

Since the year 1994, researchers have examined Jozefowicz’s claims on the relationship between neurophobia and the medical school curriculum [2–6, 8, 10–12, 15, 16, 30]. At SGU students’ neurophobia significantly increased following the neuroscience courses. Thus, methods that decrease neurophobia and increase knowledge can lead to an increase in students pursuing neurology residencies. Future studies examining the effect of various educational models on experienced neurophobia may provide further insight into ways to alter these sentiments by changes in curriculum.

## Abbreviations

CBT: Case based teaching
IRB: Insitutional Review Board
M1: First year medical student
M2: Second year medical student
PBL: Problem based learning
RAT: Readiness Assurance Test
SGU: St. George’s University
SPSS: Statistical Package for Social Sciences
TBL: Team based learning
US: United States
